# Cytotoxicity of RNase Sa to the acute myeloid leukemia Kasumi-1 cells depends on the net charge

**DOI:** 10.18632/oncoscience.97

**Published:** 2014-11-10

**Authors:** Vladimir A. Mitkevich, Ksenia M. Burnysheva, Olga N. Ilinskaya, C. Nick Pace, Alexander A. Makarov

**Affiliations:** ^1^ Engelhardt Institute of Molecular Biology, Russian Academy of Sciences, Moscow, Russia; ^2^ Department of Microbiology, Kazan Federal (Volga-Region) University, Kazan, Russia; ^3^ Department of Biochemistry and Biophysics, Texas A&M University, College Station, Texas, USA; ^4^ Department of Molecular and Cellular Medicine, Texas A&M Health Science Center, Texas A&M University, College Station, Texas, USA

**Keywords:** RNase, cytotoxicity, net charge, cationization, N-terminus, apoptosis

## Abstract

The majority of known cytotoxic RNases are basic proteins which destroy intracellular RNA. Cationization of RNases is considered to be an effective strategy for strengthening their antitumor properties. We constructed a set of RNase Sa variants consisting of charge reversal mutants, charge neutralization mutants, and variants with positively charged cluster at the N-terminus. All constructs retain a high level of catalytic activity and differ in net charge. Using acute myeloid leukemia cells Kasumi-1 we have shown that (i) cytotoxicity of RNase Sa mutants is linearly enhanced by cationization, (ii) the ability of cytotoxic mutants to induce cell death is caused by induction of apoptosis and (iii) localization of positive charge on N-terminus does not contribute to RNase Sa cytotoxicity. Capacity to induce apoptosis in malignant cells and the absence of necrotic effects make the RNase Sa mutants with high positive charge a suitable anti-cancer agent.

## INTRODUCTION

Some RNases selectively attack malignant cells, triggering apoptotic response [1-3]. Their selectivity for transformed cells is in part based on the positive charge of the enzyme, which allows it to bind anionic groups on the surface of tumor cells [1]. Tumor cells express more acidic phospholipids on the outer leaflet of their membrane than their non-tumor counterparts, and therefore are more negatively charged [4]. Most cytotoxic RNases are cationic [5]. Thus, cationization of RNases can be a promising way to enhance their antitumor effect [6]. Addition of positive charge to the variants of RNase A engineered to evade the endogenous ribonuclease inhibitor protein facilitates their entry into the cytosol, and thus increases their cytotoxicity [7]. We have previously shown that reversing five charges on the RNase from *Streptomyces aureofaciens* (RNase Sa) was sufficient to change it from one of the most acidic proteins (pI = 3.5) to one of the most basic proteins (5K Sa), and to turn a non-cytotoxic RNase into a cytotoxic one [8, 9]. However, a significant increase of the basicity of RNases could have the deleterious consequence of decreasing ribonucleolytic activity or conformational stability, which may adversely affect the cytotoxicity of the enzyme [7]. It is not clear exactly how the positive charge must be distributed over the surface of an RNase molecule for its cytotoxic properties to be optimal. For example, among 22 variants of onconase in which cationic residues were replaced with alanine it was found that a more basic variant could be either markedly more cytotoxic or less cytotoxic than a less cationic variant, depending on the distribution of its cationic residues [10].

To explore a possibility of further enhancing the cytotoxicity of RNase Sa we have constructed mutants 6K and 7K, carrying higher positive charges in comparison with 5K mutant (Table 1). All replaced residues were exposed to solvent and did not form ion pairs or hydrogen bonds. The charge neutralization mutants 3NQ and 5NQ were also constructed in order to ascertain the role of negative charge in cytotoxicity (Table 1). In these mutants the surface residues Glu and Asp, which were already replaced with lysines in the 3K and 5K mutants, have been now replaced with Gln and Asn respectively. In order to test if the net charge *per se* is responsible for the enzyme ability to attack mammalian cells or whether the distribution of charges is important as well, we have compared cytotoxicity of the RNase Sa 5K and 5NQ mutants with cytotoxicity of their variants featuring additional two Lys residues tagged to the N-terminus (Table 1). Using human acute myeloid leukemia cells Kasumi-1 we have shown that (i) cytotoxicity of RNase Sa mutants is linearly enhanced by cationization, (ii) the ability of cytotoxic mutants to induce cell death is caused by induction of apoptosis and (iii) localization of positive charge on N-terminus does not contribute to RNase Sa cytotoxicity.

**Table 1:** Net charge and pI for the surface charge reversal and charge neutralization mutants, and the N-terminal Lys-tagged variants of RNase Sa

Protein variant	Description	Net charge at pH 7^a^	pI^b^
Wild type		−7	3.5
Charge reversal mutants			
3K Sa	D1K, D17K, E41K	−1	6.4
5K Sa	3K Sa + D25K, E74K	+3	10.2
6K Sa	5K Sa + D79K	+5	>10.5
7K Sa	6K Sa + S31K	+6	>10.5
Charge neutralization mutants			
3NQ Sa	D1N, D17N, E41Q	−4	4.5
5NQ Sa	3NQ Sa + D25N, E74Q	−2	5.7
N-terminal extension variants			
K_2_^N^Sa	2K tagged to N-terminus of Sa	−5	4.9
K_2_^N^5NQ Sa	2K tagged to N-terminus of 5NQ Sa	0	8.8
K_2_^N^5K Sa	2 Lys tagged to N-terminus of 5K Sa	+5	>10.4

a The net charge at pH 7 is calculated for each variant applying model compound pK values for each ionizable residue.

b The pI values are estimates, except for the wild type, 3K and 5K, where pI have been measured [24].

## RESULTS

### Catalytic activity of RNase Sa mutants

Catalytic activity of RNase Sa mutants was determined using poly(I) as a substrate (Table 2). The greatest decrease in activity was detected for the 5K Sa and K_2_^N^5K Sa, which resulted to be 14% and 17% of the activity of the wild type enzyme, respectively. The variants 5NQ Sa and K_2_^N^5NQ Sa operate at a half of the wild type catalytic activity. The activity of all other variants is close to the activity of the wild type enzyme.

**Table 2:** Kinetic parameters characterizing the cleavage of poly(I) by RNase Sa and its variants at 25°C and pH 6.5 (0.05 M Tris, 0.05 M NaAc, 0.1 M NaCl)

RNase	k_cat_ (s^−1^)	K_M_×10^4^ (M)	k_cat_/K_M_×10^−4^ (M^−1^s^−1^)	Catalytic activity (%)^a^
Wild type	221±13	1.6±0.2	138	100
3NQ Sa	172±12	1.7±0.2	101	73
5NQ Sa	93±8	1.6±0.2	58	42
3K Sa	150±11	1.4±0.2	107	78
5K Sa	25±3	1.3±0.2	19	14
6K Sa	168±12	1.4±0.2	120	87
7K Sa	163±12	1.3±0.2	125	91
K_2_^N^Sa	238±18	1.7±0.2	140	101
K_2_^N^5NQ Sa	120±10	1.6±0.2	75	54
K_2_^N^5K Sa	36±3	1.5±0.2	24	17

a Catalytic activity of RNase Sa was taken as 100%.

### Cytotoxicity of the surface charge reversal and charge neutralization mutants of RNase Sa

**Figure 1:**
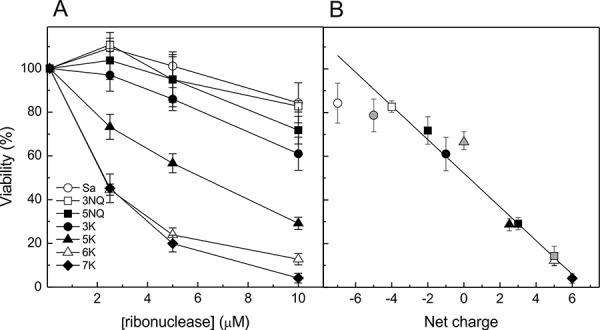
Cytotoxicity of RNase Sa and its charge reversed mutants on Kasumi-1 cells (A) Viability of Kasumi-1 cells treated 72h with different concentrations of RNase Sa (○) and its 3NQ (□), 5NQ (■), 3K (●), 5K (▲), 6K (Δ) and 7K (♦) mutants. (B) Viability of Kasumi-1 cells treated 72 h with 10 μM RNases (as specified in (A) and in Fig. 3A for K_2_^N^Sa, K_2_^N^5NQ Sa and K_2_^N^5K Sa) versus net charge on the enzymes at pH 7. This dependence can be approximated by a linear curve with the correlation coefficient R=0.97. Each value is the mean of three independent experiments with triplicate samples ± SD and is expressed as a percentage of the value obtained for the control lacking RNase.

To study cytotoxicity of the RNase Sa variants we used human acute myeloid leukemia cells Kasumi-1. We had earlier shown that these cells are highly sensitive to the toxic effect of a RNase Sa homolog binase (pI=9.5), the basic RNase from *Bacillus intermedius* [11, 12]. RNase Sa has very limited toxic effect on the Kasumi-1 cells, and at the 10 μM concentration reduces cell viability only to 84% (Fig. 1). In the group of mutants with net charges varying from −4 (3NQ Sa) up to +6 in the 7K Sa (Table 1), 7K Sa showed the highest cytotoxic effect reducing cell viability to 4% at the 10 μM concentration (Fig. 1). Concentration-response relationships (Fig. 1A) revealed IC_50_ (RNase concentration that kills 50% of cells) of 6±1 μM for the 5K Sa and 2±0.5 μM for the 6K and 7K Sa.

In the 3NQ and 5NQ RNase Sa mutants, the amino acid residues have been replaced with neutral asparagine and glutamine in the same positions as in the 3K Sa and 5K Sa. This resulted in only partial neutralization of the initial negative charge of the RNase molecule (Table 1). The cytotoxicity of these mutants, still rather negatively charged (−4 for 3NQ Sa and −2 for 5NQ Sa), практически не отличалась от wild type RNase Sa (Fig. 1A). These results show that a nearly neutral (3K Sa) or a positive net charge is a prerequisite for the RNase cytotoxicity, and that the increase of positive charge on the RNase molecule linearly correlates with the enhancement of enzyme cytotoxicity (correlation coefficient, R=0.97). This correlation is demonstrated in Fig. 1B.

**Figure 2:**
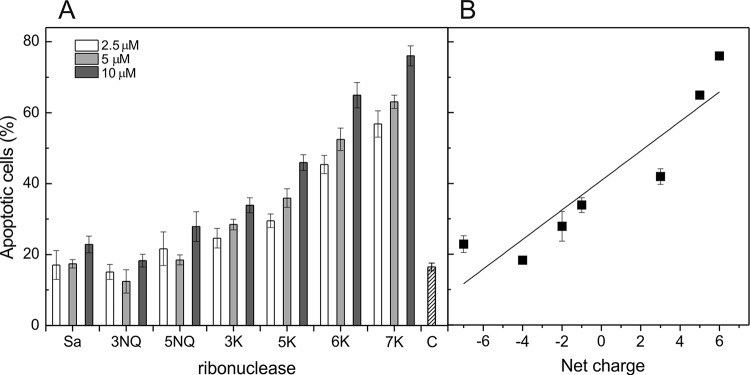
Apoptotic activity of RNase Sa and its charge reversed mutants on Kasumi-1 cells (A) Influence of RNase Sa and its mutants treatment (72 h) on the amount of apoptotic cells of Kasumi-1 cells. White columns - 2.5 μM RNase, light gray columns - 5 μM RNase and dark gray columns - 10 μM RNase. Dashed column (C) – level of apoptotic cells in the control lacking RNase. (B) The amount of apoptotic cells of Kasumi-1 cells treated 72 h with 10 μM RNases versus net charge on the enzymes at pH 7. This dependence can be approximated by a linear curve with the correlation coefficient R=0.92. The amount of apoptotic cells is expressed as a percentage of the total number of cells for each variant. Each value is the mean of three independent experiments with triplicate samples ± SD.

Using flow cytometry we have found that cytotoxic effect of RNase Sa and its mutants on Kasumi-1 cells is caused by apoptosis (Fig. 2A), with the ratio of necrotic cells in the population not above 2%. Proportion of apoptotic cells in the population treated by the RNases under study displayed linear growth with increasing positive charge on the protein molecule (R=0.92) and at 10 μM for the mutants 5K, 6K and 7K reached 46%, 65% and 76%, respectively (Fig. 2B).

### Cytotoxicity of the N-terminal Lys-tagged variants of RNase Sa

**Figure 3:**
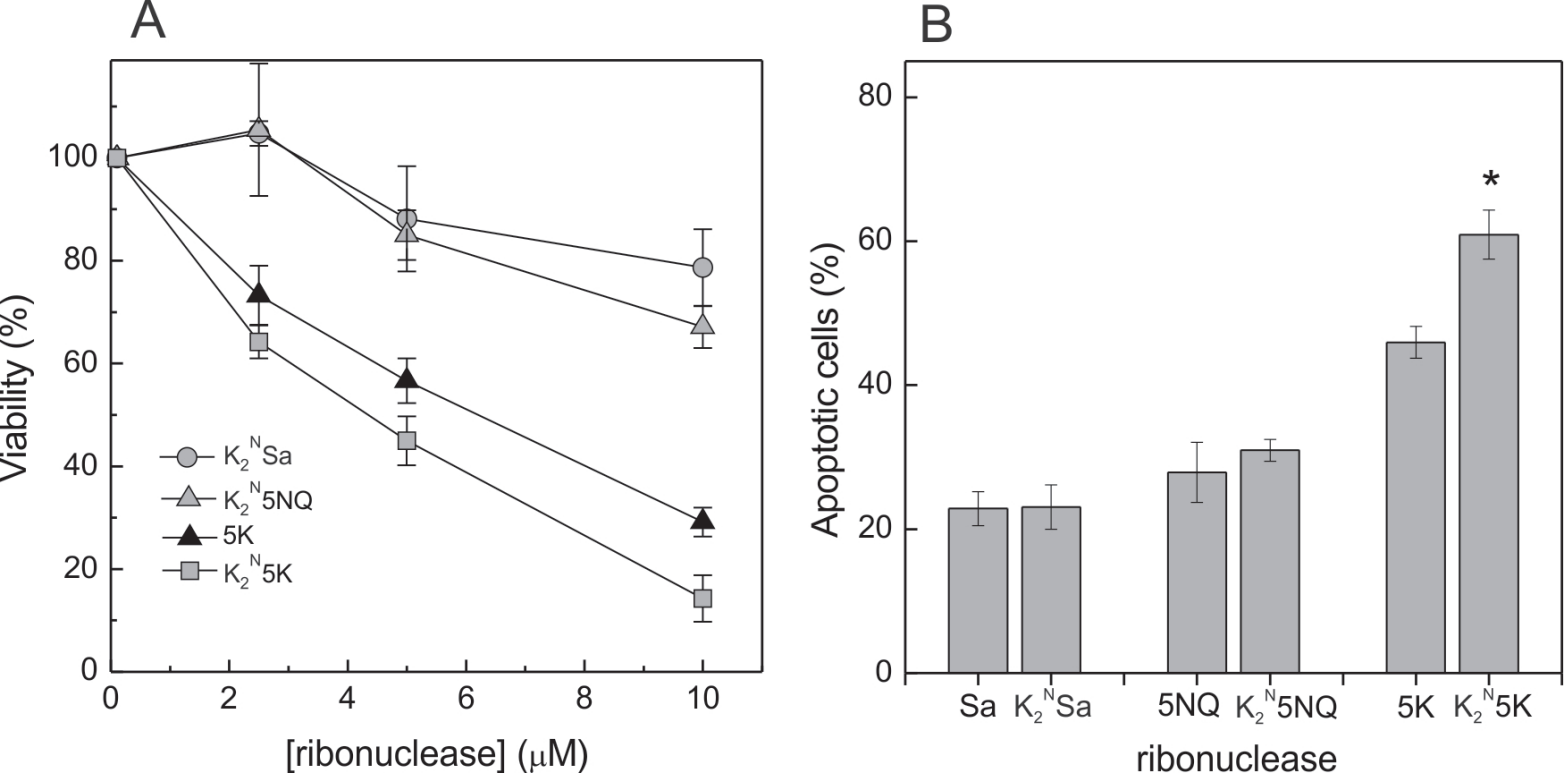
Cytotoxicity and apoptotic activity of the N-terminal Lys-tagged variants of RNase Sa (A) Viability of Kasumi-1 cells treated 72h with different concentrations of 5K RNase Sa (▲), K_2_^N^Sa (○), K_2_^N^5NQ Sa (∆) and K_2_^N^5K Sa (□). (B) Influence of 10 μM RNase Sa, 5NQ and 5K and their Lys-tagged variants (K_2_^N^Sa, K_2_^N^5NQ Sa and K_2_^N^5K Sa) on the amount of apoptotic cells of Kasumi-1 cells. Each value is the mean of three independent experiments with triplicate samples ± SD; *p<0.005.

We addressed the question of whether the location of positive charge might also affect cytotoxicity. Therefore we constructed variants with positively charged N-terminal extension groups, based on the initially non-toxic wild type RNase Sa and 5NQ Sa, and the cytotoxic 5K Sa (Table 1). Variants K_2_^N^Sa and K_2_^N^5NQ Sa did not exhibited enhanced cytotoxicity or apoptogenic activity compared to their corresponding initial forms (Figs. 1, 3). Cytotoxic properties of K_2_^N^5K Sa were higher in comparison with 5K Sa – the Kasumi-1 cell survival rate decreased faster, and the percentage of apoptotic cells in the population showed statistically significant increase (Fig. 3A,B). However cytotoxic properties of the K_2_^N^5K Sa are somewhat less prominent than that of the 6K mutant characterized by the same net charge (+5): IC_50_=4±1 μM vs 2±0.5 μM, and at 10 μM the percentage of apoptotic cells in the population reached 60.9±3.2 vs 64.9±0.6. Despite this, cytotoxicity of Lys-tagged mutants fit to a linear dependence from the charge on molecule (Fig. 1B).

## DISCUSSION

Enzymatic catalysis provides an extremely sensitive measure of native protein structure [13]. The fact that all of the RNase Sa variants have substantial catalytic activity (Table 2) suggests that their conformations are similar to that of the wild type enzyme. Decrease in activity of the 5K Sa and K_2_^N^5K Sa to 14% and 17%, respectively, is due to the Glu74Lys substitution that changes the orientation of the enzyme catalytic groups [14]. However, activity of 6K Sa and 7K Sa, also containing the Glu74Lys substitution, is close to the activity of the wild type enzyme (Table 2). Evidently, this effect is caused by the additional Asp79Lys substitution (Table 1). Activity of the Asp79Lys mutant is equal to that of the wild type Sa, although this substitution essentially influences thermal stability of the enzyme by increasing its melting temperature by 7.6°C [15]. It may be possible that stabilization of conformational mobility of the Asp79 environment upon its replacement with Lys eliminates the effect of conformational disturbances on the active center caused by the Glu74Lys substitution.

The addition of a 2 Lys tag appears to have little effect on the three-dimensional structure of wild-type RNase Sa and its 5NQ and 5K variants, based on the absence of change in the values of kcat/K_M_ for poly(I) cleavage (Table 2).

Negative surface charge of eukaryotic and bacterial cells causes their preferential interaction with cationic RNase. Membrane of tumor cells comprises more acidic phospholipids and glycoproteins compared to normal cells [16]. Cytotoxicity of the chemically modified RNase A correlates with the positive charge, reaching its limit at high values of positive charge on the molecule [17, 18]. We demonstrated earlier that the site-specific mutagenesis of the non-toxic acidic RNase Sa can be used to obtain a highly cytotoxic enzyme (5K Sa) [8]. In this work we have constructed 6K and 7K mutants of RNase Sa, in which positive net charge has been incremented to +5 and +6 (Table 1). The cytotoxicity of these mutants increased in comparison with the 5K mutant (Fig. 1). Moreover we have shown that cytotoxic properties of RNase depend linearly on its net charge (Figs. 1B and 2B). The higher was positive charge acquired by the enzyme molecule the stronger it affected cell survival rate and apoptosis. This might be important for possible application of such RNases in tumor therapy, as it would exclude adverse effects from cell death by the necrosis path.

Ostensibly, not only the net charge but its localization on the molecule contributes to the cytotoxicity of RNases. Notomista et al. [19] proposed that cytotoxic RNases must possess specific electrostatic features and structural elements serving for the interaction with cellular membranes, conceivably located at the N-terminal region of the molecule. The cluster of positively charged Lys (1,3,111,112) residues, located at the flexible coil near the N-terminus, is critical for the bactericidal activity of human RNase7 [20, 21]. Main contribution to the toxicity of the eosinophil cationic protein is made by a region located at the protein N-terminus, residues 11- 35 [22]. The variant of bovine seminal RNase (BS-RNase), G38K-BSRNase, carrying enhanced cluster of positive charges at the N-terminus surface, possesses an increased cytotoxicity toward tumor cells [23]. However, cationization of the N-terminus of RNase Sa (K_2_^N^Sa) and the charge neutralization mutant (K_2_^N^5NQ Sa) appeared to be ineffective (Fig. 3). It should be noted that cytotoxicity of the 3K Sa is virtually identical to cytotoxicity of K_2_^N^5NQ Sa (Figs. 1, 3), although the charge of the first mutant is negative (net charge −1), while for the second mutant it is neutral (net charge 0). Further, the 6K mutant had even stronger effect on the survival rate and apoptosis induction in the Kasumi-1 cells compared to the mutant K_2_^N^5K Sa with similar net charge (+5) (Figs. 1-3). These results suggest that an important factor defining the RNase Sa cytotoxicity is the distribution of positive charge on the molecular surface rather than its localization on the N-terminus. This is confirmed by the evidence that cytotoxicity of the RNase Sa mutants with substitutions of residues located on molecular surface and of the Lys-tagged variants fits linear curve against the molecular charge (Fig. 1B).

The mechanism of antitumor activity of exogenous RNase remains largely unclear, but the contribution of positive charge of the molecule to RNase-induced cell death is beyond doubt [2, 3]. Cationization of RNases using chemical agents is a common approach to enhancing their cytotoxicity. However, to separate cytotoxic effects of the actual RNases from the effects of associated polycations, the latter should not constitute a significant part of the chimeric molecule. The set of RNase Sa mutants and their variants with the N-terminal extension developed by us, allowed to clarify the role of positive net charge and its molecular distribution in cytotoxicity. Here we have shown that cytotoxic properties of these RNases increase linearly with the net charge growth, and that the charge localization on the N-terminus does not provide additional contribution to the cytotoxicity. Cytotoxicity of the RNase Sa mutants to Kasumi-1 cells is caused by their apoptosis-inducing effect, which allows to consider them as potential anti-cancer agents along with other RNases.

## METHODS

### Proteins

RNase Sa and the mutants listed above were prepared, expressed, and purified as described previously [15, 24].

### Catalytic activity

Catalytic activity was determined for the poly(I) hydrolysis at 25°C, as described in [14]. The initial rates were measured by recording the change in absorbance at 248 nm. The buffer used was 0.05 M Tris, 0.1 M sodium chloride, and 0.05 M sodium acetate, pH 6.5. Concentrations of RNase Sa and its mutants were determined spectrophotometrically using the same molar extinction coefficient ε_278_ = 12300 M^−1^ cm^−1^ [25].

### Cell culture

Human acute myeloid leukemia cells Kasumi-1, obtained from the Heinrich-Pette Institute Leibniz Institute for Experimental Virology (Hamburg, Germany), were used. Kasumi-1 cells were grown on RPMI-1640 media containing 20 % FCS, 100 units/ml penicillin, 100 μg/ml streptomycin, and 1 mM sodium pyruvate at 37°C in humid atmosphere with 5 % CO_2_.

### Cell Viability

Cellular viability was assessed with a WST-1-based test (Roche Diagnostics) as described earlier [11]. The cells were plated into 96-well plates (3 × 10^4^ cells per well) and cultured for 24 h at 37 °C. Then the cells were treated with binase. After 72 h of the binase treatment the cells were incubated with the WST-1 reagent for 60 min at 37 °C. The absorbance of samples was measured with an Anthos 2020 microplate reader (Anthos Labtech Instruments GmbH) at 450 nm. The reference wavelength was 620 nm. A mixture of cell-free medium with the WST-1 reagent was used as a background control. The viability of untreated cells was taken as 100 %. All reported values are means of three independent measurements with triplicate samples ± standard deviations.

### Determination of the amounts of apoptotic and necrotic cells by flow cytometry

Apoptotic and necrotic cells in population were determined by double staining with Annexin V-FITC (Molecular Probes) [26] and propidium iodide (PI) (Sigma) [27]. Annexin positive cells considered as apoptotic while PI positive Annexin negative cells considered as necrotic. All measurements were carried out on a GALLIOS flow cytometer (Beckman Coulter).

### Statistical analysis

Values are shown as means ± standard deviations. The comparison of data groups was performed using Student's t-test; p < 0.05 was considered significant.

## Abbreviations

RNase Sa: ribonuclease from Streptomyces aureofaciens
BS-RNase: bovine seminal RNase
